# Evaluation of Mobile Applications as an Alternative to Weighed Food Records and Food Frequency Questionnaires for Dietary Assessment in Japan

**DOI:** 10.7759/cureus.87725

**Published:** 2025-07-11

**Authors:** Junko Nohara, Tatsuya Koyama

**Affiliations:** 1 Department of Nutrition, Kio University, Koryo, JPN; 2 Department of Nutritional Guidelines, National Institutes of Biomedical Innovation, Health, and Nutrition, Ibaraki, JPN

**Keywords:** dietary assessment, digital health tools, food frequency questionnaire (ffq), mobile applications, nutritional epidemiology in japan, weighed food records

## Abstract

Introduction

Dietary surveys are essential in nutritional epidemiology. While the weighed food record method is highly accurate and commonly used in Japan, it is unsuitable for large-scale studies due to its labor-intensive nature. Mobile applications for dietary assessment have gained popularity as alternatives, offering convenience and automation. This study aimed to evaluate the feasibility of using mobile applications as a substitute for traditional methods like weighed food records and food frequency questionnaires (FFQs) in population-based dietary assessments, specifically by examining the correlation between nutrient intake estimates obtained from a mobile application and those from traditional methods.

Methods

This cross-sectional study was conducted from May 1 to June 30, 2022, and included 85 third-year female students (mean age: 20.2 ± 0.6 years; range: 20-25 years) from the Department of Nutrition, Faculty of Health Sciences, Kio University, Nara, Japan. All participants were Japanese nationals with a uniform educational background, were unmarried, and had received formal training in nutrition and dietary assessment as part of their curriculum.

Dietary intake data were collected using both weighed food records and the Calomeal® mobile application (Life Log Technology, Inc., Tokyo, Japan), which enables users to record intake via food photos or manual entry and provides automatic analysis for 29 nutrients. Each participant recorded all foods and beverages consumed on a single weekday of their choice during the study period. There were no dropouts; all enrolled participants completed the study procedures.

Nutrient intake estimates from the app and weighed food records were compared using Spearman’s correlation coefficients. The proportion of participants not meeting the Estimated Average Requirement (EAR) was assessed using the cut-point method. All statistical analyses were performed using IBM SPSS Statistics for Windows, version 28 (IBM Corp., Armonk, New York, United States).

Results

High correlations (0.7 ≤ |ρ| ≤ 1) were observed between nutrient intake estimates from the app and weighed food records for most nutrients, although moderate correlations (0.4 ≤ |ρ| < 0.7) were noted for specific nutrients like magnesium, iron, and vitamin B12 during certain meals. Comparisons with FFQs showed weaker correlations, consistent with previous findings. The mobile application demonstrated significant potential as a reliable tool for dietary assessment, particularly in large-scale studies where traditional methods pose logistical challenges.

Conclusions

Mobile applications like Calomeal® provide a viable alternative to weighed food records for dietary assessments, offering high accuracy and ease of use. They outperform FFQs in precision and reduce logistical challenges, making them suitable for large-scale nutritional studies.

## Introduction

Dietary surveys play a crucial role in nutritional epidemiology. The 24-hour recall method is widely used internationally [1,2], whereas the weighed food record method is the primary dietary survey approach employed by the Japanese government to assess the nutritional status of the population [3]. Although the weighed food record method is recognized for its high accuracy, it is deemed unsuitable for large-scale studies because of its labor-intensive and time-consuming nature [4]. Consequently, food frequency questionnaires (FFQs) are frequently used, albeit with lower accuracy than other methods.

Recently, numerous mobile applications have been developed to automatically analyze nutritional status based on user-uploaded photos. Many individuals prefer these applications for assessing their dietary intake over traditional self-reporting methods, such as weighed food records [5-7]. A study evaluating five major mobile food record applications in Japan demonstrated a strong correlation with weighed food records regarding energy and nutrient intake estimates [8].

In large-scale nutritional epidemiological studies in Japan, group nutritional assessments are currently based on the proportion of individuals whose dietary intake falls below or above the estimated average requirement (EAR), as outlined in the Dietary Reference Intakes (DRI) for Japanese (2020 edition) [9]. Therefore, this study aimed to investigate whether dietary intake data obtained via mobile applications can serve as a viable alternative to weighed food records and FFQs for population-based assessments, by specifically analyzing the correlation between nutrient intake estimates from the mobile application and those from traditional methods.

## Materials and methods

This was a cross-sectional study conducted from May 1 to June 30, 2022, at Kio University, Koryo, Nara, Japan. The study was approved by the Research Ethics Review Board of Kio University (approval number: r4-09), and conducted in accordance with the guidelines of the Declaration of Helsinki.

Study population

The participants were selected from among female students who had received training in weighing food records and were currently enrolled in the third year of the dietitian training program at the Department of Nutrition, Faculty of Health Sciences, Kio University, ensuring a consistent education level across the cohort. More than 90% of students in the Department of Nutrition were female. Therefore, only female students were included in the study to ensure that the sample was representative of the actual student population. All participants were Japanese nationals and were unmarried. Physical activity levels among participants ranged from low to moderate. With respect to nutrition knowledge and lifestyle, all participants had received formal training in nutrition and dietary assessment as part of their curriculum. Only those who provided informed consent to participate were included. Information on individual or household income was not collected, as the study focused on a homogeneous group of full-time university students who are presumed to have similar socioeconomic backgrounds.

Sample size determination**

All eligible female students were invited to participate, and those who provided informed consent were included, resulting in a final sample size of 85 participants. While a formal power analysis was not conducted, the sample size of 85 was considered sufficient to detect moderate to high correlations (e.g., ρ≥0.4) between dietary assessment methods, based on previous validation studies in similar settings. This rationale is supported by systematic reviews in the field. Zhang et al., for example, reported that sample sizes of several dozen to around 100 participants are commonly used and provide reliable results in validation studies of dietary record apps [10].

Sampling technique

Convenience sampling was used for participant recruitment. All eligible third-year female students in the department during the study period were invited to participate, and those who consented were included in the study. This non-probability sampling method was chosen to ensure that the sample was representative of the actual cohort within the department, but it may introduce selection bias and limit the generalizability of the findings beyond the specific cohort studied.

Selection and rationale for the mobile application

We selected Calomeal® (Life Log Technology, Inc., Tokyo, Japan) as the most suitable mobile dietary survey application for this study from among five major apps previously evaluated for their capacity to estimate energy and nutrient intakes in population-based settings [8]. The selection criteria included accessibility for the general public, high correlation with weighed dietary records, user-friendliness, data visualization capabilities, and the ability to record and analyze detailed nutrient information suitable for population assessment.

Calomeal is a Japanese mobile dietary assessment application that enables users to record their dietary intake either by uploading food photos or manually searching from a comprehensive database of over 26,000 food items. Designed for public accessibility, it boasts over four million users in Japan. The app provides automatic nutritional analysis for 29 nutrients and visualizes daily intake for energy and key nutrients. Its core features are available free of charge, making it accessible to the general public and suitable for large-scale surveys [11].

While premium features are available at a modest monthly fee, all features required for this study, including dietary recording and nutrient analysis, were accessible in the free version. Accordingly, all analyses in this study were conducted using the free version of the application, ensuring that no participant incurred any cost.

Previous research has demonstrated the effectiveness of Calomeal and similar mobile applications in Japan. For example, Shinozaki and Murakami reported moderate to high correlations (ρ=0.60-0.85) between app-based and traditional weighed food records for energy and nutrient intakes at the population level [8]. In addition, recent clinical studies have further validated the utility of Calomeal in health management settings. Kusano et al. demonstrated the effectiveness of dietary counseling using Calomeal for patients with nonalcoholic fatty liver disease, highlighting its practical application in clinical interventions [12]. Similarly, Tsunemi et al. conducted a pilot study using Calomeal for dietary management in patients with type 2 diabetes, reporting significant improvements in glycemic control and weight management among participants [11]. These findings collectively support the use of Calomeal as a reliable tool for dietary assessment and intervention across diverse settings.

Dietary data collection

All participants completed both the weighed food record and the mobile application photo record on a single weekday of their choice during the study period. The dietary assessment was conducted for one weekday only, selected at the participant’s discretion within the designated period. There were no dropouts; all enrolled participants completed the study procedures.

Weighed Food Record

A research dietitian instructed the participants on how to maintain a food record. They were asked to select a weekday and record the weights of all foods and beverages consumed on that day using scales, measuring spoons, and cups. The main items recorded included (i) the name of the dish, (ii) the name of the food (including ingredients in mixed dishes), and (iii) the weight or approximate quantity of food and beverages. Research dietitians collected and verified the data.

Each food item was assigned a code based on the Standard Tables of Food Composition in Japan (STFCJ), 8th edition [13]. For foods consumed outside or purchased, dietitians estimated the ingredient weights as accurately as possible using information from restaurant websites, ingredient labels, nutritional information on packaging, and cookbooks. Daily energy and nutrient intakes from food and beverages, excluding supplements, were calculated for each participant using STFCJ data.

Participants were asked to provide additional information when necessary to clarify the food names and quantities listed on their forms. All food codes and weights were double-checked by two dieticians.

Mobile Application

Participants took photographs of all foods and beverages consumed. Dietitians then entered the dietary data into the mobile application using these photographs and the participants’ weighed food records, following a standardized procedure that simulated typical app usage. The app facilitated intuitive food selection based on photos taken by the participants, allowing them to choose items that closely resembled the items in their images. Portion sizes were adjusted within the application to best reflect the net weight recorded in weighed food records.

Comparison with FFQ

In addition to using the app, dietary assessments were compared with a larger FFQ consisting of 172 items developed for the Japan Public Health Center-based prospective Study for the Next Generation (JPHC-NEXT) [14]. Food and nutrient intakes were calculated using dedicated software (FFQ NEXT, Kenpakusha, Tokyo, Japan) by referring to the STFCJ 8th edition [13].

Data analysis

The energy and nutrient estimates derived from the weighed food records, app, and FFQ were evaluated against the standard intakes defined by the Japanese DRI [15], using the STFCJ [13] as a reference. The proportion of estimated nutrient values from the app, dietary records, and FFQ was analyzed using Spearman’s correlation coefficient.

The DRI includes various nutrient indicators that serve multiple purposes. In particular, the EAR represents "the amount that meets the nutritional needs of 50% of the population" and is used to assess nutritional deficiencies in the population. In this study, the proportion of inadequate intake was assessed based on the percentage of participants who did not meet the EAR using the cut-point method [16]. Kappa statistics, chi-squared tests, and McNemar’s test were used to assess agreement between methods (above or below EAR).

Statistical significance was set at P <0.05. All statistical analyses were performed using IBM SPSS Statistics for Windows, version 28 (IBM Corp., Armonk, New York, United States).

## Results

A total of 85 female university students were included in the study. They had a mean age of 20.2 years (SD: 0.6), a mean height of 159.0 cm (SD: 5.5), a mean weight of 50.8 kg (SD: 6.6), and a mean BMI of 20.1 kg/m² (SD: 2.1). The basic characteristics of the participants are presented in Table 1.

**Table 1:** Basic characteristics of the study participants (N=85)

Variables	Unit	Mean	SD
Age	years	20.2	0.6
Height	cm	159.0	5.5
Weight	kg	50.8	6.6
BMI	kg/m^2^	20.1	2.1

Table 2 presents a detailed comparison of the nutrient intakes estimated by the food weighing method and mobile application for each meal (breakfast, lunch, dinner, and snacks). For most nutrients, the Spearman correlation coefficients between the two methods were high (ρ≥ 0.7), indicating a strong agreement. For example, the correlation for calcium intake at breakfast was ρ=0.882 (95%CI: 0.823-0.922). However, moderate correlations (0.4 ≤ ρ < 0.7) were observed for magnesium, iron, and vitamin B12 at lunch, for magnesium, vitamin B1, vitamin B6, and vitamin C at dinner, and for vitamin C in snacks. These results suggest that although the app generally performs well, there may be limitations in estimating certain micronutrients in specific meals.

**Table 2:** Comparison of daily nutrient intakes estimated by the food weighing method and mobile application (N=85)

Variables		Unit	Weighed Food Records		Mobile Application		Correlation coefficient	
			Mean	SD	Mean	SD	ρ	95%CI
Breakfast	Energy intake	kcal/day	304	140	336	161	0.845	(0.771-0.897)
	Protein intake	g/day	11.7	20.4	11.9	8.0	0.848	(0.774-0.898)
	Calcium intake	mg/day	134	109	144	125	0.882	(0.823-0.922)
	Magnesium intake	mg/day	42	32	43	33	0.849	(0.776-0.899)
	Iron intake	mg/day	1.4	3.6	1.2	1.2	0.823	(0.740-0.882)
	Retinol activity equivalent intake	μgRAE/day	66	88	89	98	0.815	(0.729-0.876)
	Vitamin B_1_ intake	mg/day	0.12	0.08	0.15	0.13	0.743	(0.629-0.825)
	Vitamin B_2 _intake	mg/day	0.21	0.16	0.23	0.19	0.886	(0.829-0.924)
	Vitamin B_6 _intake	mg/day	0.18	0.19	0.19	0.20	0.791	(0.695-0.859)
	Vitamin B_12 _intake	mg/day	0.6	1.6	0.8	2.4	0.812	(0.724-0.874)
	Vitamin C intake	mg/day	15	24	14	21	0.811	(0.722-0.873)
Lunch	Energy intake	kcal/day	462	181	491	201	0.755	(0.646-0.834)
	Protein intake	g/day	14.5	8.7	18.4	11.2	0.779	(0.679-0.851)
	Calcium intake	mg/day	87	77	71	53	0.714	(0.591-0.805)
	Magnesium intake	mg/day	47	26	47	26	0.566	(0.401-0.695)
	Iron intake	mg/day	1.6	0.8	1.6	0.9	0.646	(0.502-0.755)
	Retinol activity equivalent intake	μgRAE/day	135	133	115	168	0.717	(0.594-0.807)
	Vitamin B_1_ intake	mg/day	0.24	0.21	0.24	0.24	0.725	(0.605-0.813)
	Vitamin B_2 _intake	mg/day	0.27	0.19	0.26	0.20	0.827	(0.745-0.884)
	Vitamin B_6 _intake	mg/day	0.27	0.18	0.27	0.20	0.737	(0.621-0.821)
	Vitamin B_12 _intake	mg/day	0.8	0.8	0.9	1.0	0.646	(0.503-0.756)
	Vitamin C intake	mg/day	20	22	16	20	0.807	(0.717-0.870)
Dinner	Energy intake	kcal/day	527	179	560	182	0.718	(0.596-0.807)
	Protein intake	g/day	21.6	9.8	28.1	12.9	0.734	(0.618-0.819)
	Calcium intake	mg/day	113	75	116	83	0.720	(0.599-0.809)
	Magnesium intake	mg/day	76	32	81	39	0.658	(0.517-0.764)
	Iron intake	mg/day	2.4	1.1	2.6	1.4	0.721	(0.600-0.810)
	Retinol activity equivalent intake	μgRAE/day	160	135	150	132	0.744	(0.631-0.826)
	Vitamin B_1_ intake	mg/day	0.35	0.24	0.37	0.26	0.555	(0.388-0.687)
	Vitamin B_2 _intake	mg/day	0.36	0.18	0.37	0.22	0.787	(0.690-0.857)
	Vitamin B_6 _intake	mg/day	0.48	0.26	0.50	0.28	0.656	(0.515-0.762)
	Vitamin B_12 _intake	mg/day	1.8	2.5	2.6	4.2	0.758	(0.650-0.836)
	Vitamin C intake	mg/day	26	18	24	17	0.642	(0.497-0.753)
Snack	Energy intake	kcal/day	121	118	135	137	0.885	(0.828-0.924)
	Protein intake	g/day	2.7	3.1	3.0	3.4	0.854	(0.783-0.903)
	Calcium intake	mg/day	62	85	61	78	0.757	(0.648-0.835)
	Magnesium intake	mg/day	15	20	15	21	0.800	(0.708-0.866)
	Iron intake	mg/day	0.4	0.6	0.9	4.0	0.792	(0.696-0.860)
	Retinol activity equivalent intake	μgRAE/day	34	52	39	89	0.815	(0.729-0.876)
	Vitamin B_1_ intake	mg/day	0.04	0.05	0.05	0.08	0.763	(0.656-0.839)
	Vitamin B_2 _intake	mg/day	0.10	0.12	0.09	0.13	0.792	(0.696-0.860)
	Vitamin B_6 _intake	mg/day	0.05	0.08	0.06	0.12	0.741	(0.626-0.824)
	Vitamin B_12 _intake	mg/day	0.1	0.2	0.1	0.2	0.801	(0.709-0.866)
	Vitamin C intake	mg/day	8	38	12	66	0.561	(0.396-0.692)

Table 3 shows the mean (SD) nutrient intakes and the prevalence of inadequacy based on the EAR for both methods. The prevalence of protein inadequacy was 28.2% (food weighing) and 15.3% (mobile app). For calcium, the prevalence of inadequacy was high for both methods (food weighing: 83.5%, mobile app: 82.4%). Correlation coefficients for inadequacy rates between methods were high for most nutrients (e.g., protein ρ = 0.70, calcium ρ = 0.81), indicating consistency in classifying participants as above or below the EAR.

**Table 3:** Nutrient intakes and prevalence of EAR inadequacy assessed by the food weighing method and mobile application (N=85) EAR: estimated average requirement

Variables	Unit	Reference Values		Weighed Food Records		EAR Inadequacy (%)	Mobile Application		EAR Inadequacy (%)	Correlation Coefficient	
		EAR	Coefficient of variation	Mean	SD		Mean	SD		ρ	95%CI
Protein intake	g/day	40	12.5%	50.4	27.4	28.2	61.4	20.6	15.3	0.696	(0.605-0.769)
Calcium intake	mg/day	550	10%	397	178	83.5	391	175	82.4	0.806	(0.743-0.855)
Magnesium intake	mg/day	230	10%	180	59	85.9	187	64	77.6	0.730	(0.647-0.796)
Iron intake	mg/day	8.5	10%	5.7	4.1	76.5	6.2	4.4	62.4	0.603	(0.492-0.695)
Retinol activity equivalent intake	μgRAE/day	450	20%	395	217	57.6	391	255	68.2	0.704	(0.614-0.775)
Vitamin B_1_ intake	mg/day	0.9	10%	0.75	0.35	75.3	0.81	0.40	67.1	0.726	(0.642-0.793)
Vitamin B_2 _intake	mg/day	1.0	10%	0.94	0.35	56.5	0.96	0.42	56.5	0.826	(0.768-0.870)
Vitamin B_6 _intake	mg/day	1.0	10%	0.98	0.40	60.0	1.03	0.43	49.4	0.731	(0.649-0.797)
Vitamin B_12 _intake	mg/day	2.0	10%	3.4	3.1	41.2	4.4	5.0	36.5	0.759	(0.683-0.819)
Vitamin C intake	mg/day	85	10%	69	55	76.5	67	76	74.1	0.771	(0.699-0.828)

The correlation coefficients for participants who did not meet the DRIs were high (≥ 0.6) for all nutrients. The inadequacy of nutrient intake is summarized in Table 4. Nutrient intake adequacy was assessed by comparing the usual intake with age- and sex-specific reference intakes in the Japanese DRI [15]. Data from weighed food records and mobile app datasets were assessed for consistency in two categories: "above EAR" and "below EAR.” McNemar's test was used in addition to kappa statistics and chi-squared tests. The results of McNemar's test indicated significant differences in protein levels (p<0.05). There were no statistically significant differences between the methods for any of the other nutrients. With regard to the kappa-statistic agreement rates, iron showed very low agreement (kappa = -0.09), suggesting poor consistency between the two methods for this nutrient measurement.

**Table 4:** Cross-classification and agreement between the food weighing method and mobile application for assessing EAR inadequacy using Kappa statistics, McNemar’s test, and Chi-square test (N=85) EAR: estimated average requirement

Variables	Types of food records	Category	Mobile Application		McNemar	Kappa coefficient	95%CI	Agreement (%)
			Less than EAR	More than EAR	p-value			
Protein	Food weighing method	Less than EAR	13	11	<0.001	0.629	(0.425-0.834)	87.1
		More than EAR	0	61				
Calcium		Less than EAR	64	7	1.000	0.460	(0.082-0.694)	84.3
		More than EAR	6	6				
Magnesium		Less than EAR	62	11	0.118	0.415	(0.146-0.684)	82.4
		More than EAR	4	8				
Iron		Less than EAR	68	12	0.143	-0.091	(0.554-0.373)	80.0
		More than EAR	5	0				
Vitamin A		Less than EAR	44	5	0.064	0.527	(0.339-0.714)	77.6
		More than EAR	14	22				
Vitamin B1		Less than EAR	51	13	0.167	0.460	(0.246-0.674)	77.6
		More than EAR	6	15				
Vitamin B2		Less than EAR	41	7	1.000	0.665	(0.505-0.825)	83.5
		More than EAR	7	30				
Vitamin B6		Less than EAR	35	16	0.093	0.460	(0.272-0.649)	72.9
		More than EAR	7	27				
Vitamin B12		Less than EAR	26	9	0.424	0.654	(0.488-0.820)	83.5
		More than EAR	5	45				
Vitamin C		Less than EAR	56	9	0.804	0.494	(0.271-0.718)	81.2
		More than EAR	7	13				

Table 5 compares the nutrient intakes estimated using the food weighing method, mobile application, and FFQ. The correlation coefficients between the food weighing method and mobile app were high for all nutrients (e.g., iron, ρ=0.712; calcium, ρ=0.806; magnesium, ρ=0.730; vitamin B2, ρ=0.826). In contrast, the correlations between the food weighing method and the FFQ were low only for calcium (ρ=0.317), magnesium (ρ=0.299), iron (ρ=0.262), and vitamin B2 (ρ=0.253), and no significant correlations were observed for the other nutrients.

**Table 5:** Comparison of nutrient intakes estimated by the food weighing method, mobile application, and FFQ (N=85) FFQ: food frequency questionnaire

Variables	Unit	Weighed Food Records		Mobile Application		FFQ		Correlation Coefficient		Correlation Coefficient		Correlation Coefficient	
								Food weight VS Mobile application		Food weight VS FFQ		Mobile Application VS FFQ	
		Mean	SD	Mean	SD	Mean	SD	ρ	95%CI	ρ	95%CI	ρ	95%CI
Energy intake	kcal/day	1415	322	1521	360	1647	454	0.724	(0.604-0.812)	0.106	(-0.116-0.318)	0.043	(-0.171-0.254)
Protein intake	g/day	50.4	27.4	61.4	20.6	60.5	19.5	0.685	(0.553-0.784)	0.095	(-0.127-0.307)	0.145	(-0.070-0.348)
Calcium intake	mg/day	397	178	391	175	463	245	0.806	(0.716-0.870)	0.317	(0.105-0.501)	0.453	(0.266-0.608)
Magnesium intake	mg/day	180	59	187	64	226	81	0.730	(0.612-0.816)	0.299	(0.086-0.486)	0.392	(0.195-0.558)
Iron intake	mg/day	5.7	4.1	6.2	4.4	7.0	2.6	0.712	(0.588-0.803)	0.262	(0.046-0.456)	0.220	(0.007-0.414)
Retinol activity equivalent intake	μgRAE/day	395	217	391	255	604	447	0.704	(0.577-0.797)	0.174	(-0.047-0.378)	0.014	(-0.200-0.227)
Vitamin B_1_ intake	mg/day	0.75	0.35	0.81	0.40	0.84	0.28	0.727	(0.608-0.814)	0.145	(-0.077-0.353)	0.202	(-0.011-0.398)
Vitamin B_2 _intake	mg/day	0.94	0.35	0.96	0.42	1.13	0.51	0.826	(0.743-0.883)	0.253	(0.036-0.448)	0.276	(0.066-0.462)
Vitamin B_6 _intake	mg/day	0.98	0.40	1.03	0.43	1.12	0.38	0.731	(0.614-0.817)	0.188	(-0.032-0.391)	0.205	(-0.008-0.401)
Vitamin B_12 _intake	mg/day	3.4	3.1	4.4	5.0	4.6	2.6	0.763	(0.657-0.840)	-0.045	(-0.262-0.176)	-0.031	(-0.242-0.184)
Vitamin C intake	mg/day	69	55	67	76	86	46	0.769	(0.665-0.844)	0.149	(-0.073-0.356)	0.188	(-0.026-0.386)

Table 6 examines the associations between average usual nutrient intake and the prevalence of inadequacy across the three dietary assessment methods. The correlation coefficients between the food weighing method and the mobile application were generally high for most nutrients, indicating strong consistency in identifying participants with inadequate intakes (e.g., protein: ρ=0.696, calcium: ρ=0.806, and magnesium: ρ=0.730). In contrast, the associations between the food weighing method and the FFQ were weak for all nutrients, with low correlation coefficients observed for calcium (ρ=0.304) and no significant correlations for the other nutrients. These results suggest that the mobile application provides estimates of inadequacy prevalence that are more comparable to the food weighing method, whereas the FFQ shows almost no agreement with it.

**Table 6:** Associations between average nutrient intakes estimated by different dietary assessment methods and prevalence of EAR inadequacy (N=85) EAR: estimated average requirement

Variables	Unit	Reference Values		EAR Inadequacy (Weighed food Records), n (%)	EAR Inadequacy (Mobile Application), n (%)	EAR Inadequacy (FFQ), n (%)	Correlation Coefficient		Correlation Coefficient		Correlation Coefficient			
							Food weight VS FFQ		Mobile application VS FFQ		Food weight VS Mobile application			
		EAR	Coefficient of variation				ρ	95%CI	ρ	95%CI	ρ	95%CI		
Protein intake	g/day	40	12.5%	24 (28.2)	13 (15.3)	9 (10.6)	0.094	(-0.128-0.307)	0.165	(-0.05-0.365)	0.696	(0.605	-	0.769)
Calcium intake	mg/day	550	10%	71 (83.5)	70 (82.4)	61 (71.8)	0.304	(0.091-0.491)	0.456	(0.269-0.610)	0.806	(0.743	-	0.855)
Magnesium intake	mg/day	230	10%	73 (85.9)	66 (77.6)	49 (57.6)	0.299	(0.085-0.486)	0.394	(0.197-0.560)	0.730	(0.647	-	0.796)
Iron intake	mg/day	8.5	10%	80 (94.1)	73 (85.9)	62 (72.9)	0.261	(0.045-0.454)	0.235	(0.023-0.427)	0.603	(0.492	-	0.695)
Retinol activity equivalent intake	μgRAE/day	450	20%	49 (57.6)	41 (68.2)	41 (68.2)	0.176	(-0.045-0.381)	0.012	(-0.202-0.225)	0.704	(0.614	-	0.775)
Vitamin B_1_ intake	mg/day	0.9	10%	64 (75.3)	57 (67.1)	54 (63.5)	0.142	(-0.079-0.351)	0.195	(-0.019-0.392)	0.726	(0.642	-	0.793)
Vitamin B_2 _intake	mg/day	1.0	10%	48 (56.5)	48 (56.5)	42 (49.4)	0.253	(0.036-0.448)	0.271	(0.062-0.458)	0.826	(0.768	-	0.870)
Vitamin B_6 _intake	mg/day	1.0	10%	51 (60.0)	42 (49.4)	35 (41.2)	0.19	(-0.031-0.393)	0.208	(-0.006-0.403)	0.731	(0.649	-	0.797)
Vitamin B_12 _intake	mg/day	2.0	10%	35 (41.2)	31 (36.5)	8 (9.4)	-0.07	(-0.285-0.152)	-0.095	(-0.302-0.120)	0.759	(0.683	-	0.819)
Vitamin C intake	mg/day	85	10%	65 (76.5)	63 (74.1)	47 (55.3)	0.15	(-0.072-0.357)	0.195	(-0.019-0.392)	0.771	(0.699	-	0.828)

Table 7 shows the agreement between the FFQ and the weighed meal records, and Table 8 shows the agreement between the FFQ and the mobile application in classifying participants as above or below the EAR. Both tables indicate that the agreement was low for magnesium and vitamin B2 intake. The agreement rate between the FFQ and the food weighing method for magnesium was 64.7% (kappa=0.207), and for vitamin B2, it was 62.4% (kappa=-0.248). Additionally, the agreement between the FFQ and the mobile application was 72.9% (kappa=0.247) for calcium, 65.9% (kappa=0.255) for magnesium, and 64.7% (kappa=0.295) for vitamin B2. For the other nutrients, there was little to no agreement, as reflected by the very low or negative kappa values. These results suggest that the FFQ performs poorly in classifying individuals’ nutrient adequacy status compared with weighed meal records and mobile applications, particularly for micronutrients.

**Table 7:** Agreement between FFQ and food weighing method for assessing EAR inadequacy using Kappa statistics and cross-classification (N=85) FFQ: food frequency questionnaire; EAR: estimated average requirement

Variables	Types of food records	Category	FFQ method		McNemar	Kappa coefficient	95%CI	Agreement (%)
			Less than EAR	More than EAR	p-value			
Protein	Food weighing method	Less than EAR	4	20	0.004	0.105	(-0.190-0.399)	70.6
		More than EAR	5	56				
Calcium		Less than EAR	53	18	0.076	0.136	(-0.141-0.413)	69.4
		More than EAR	8	6				
Magnesium		Less than EAR	46	27	＜0.001	0.207	(-0.021-0.435)	64.7
		More than EAR	3	9				
Iron		Less than EAR	59	21	＜0.001	0.051	(-0.270-0.373)	71.8
		More than EAR	3	2				
Vitamin A		Less than EAR	25	24	0.268	0.064	(-0.147-0.275)	52.9
		More than EAR	16	20				
Vitamin B1		Less than EAR	43	21	0.110	0.128	(-0.111-0.366)	62.4
		More than EAR	11	10				
Vitamin B2		Less than EAR	29	19	0.377	0.248	(0.043-0.454)	62.4
		More than EAR	13	24				
Vitamin B6		Less than EAR	24	27	0.014	0.136	(-0.068-0.341)	55.3
		More than EAR	11	23				
Vitamin B12		Less than EAR	2	33	＜0.001	-0.071	(-0.318-0.176)	54.1
		More than EAR	6	44				
Vitamin C		Less than EAR	37	28	0.005	0.053	(-0.171-0.277)	55.3
		More than EAR	10	10				

**Table 8:** Agreement between food frequency questionnaire and mobile application for assessing EAR inadequacy using Kappa statistics and cross-classification (N=85) EAR: estimated average requirement

Variables	Types of food records	Category	Mobile application method		McNemar	Kappa coefficient	95%CI	Agreement (%)
			Less than EAR	More than EAR	p-value			
Protein	FFQ method	Less than EAR	2	7	0.480	0.065	(-0.319-0.448)	78.8
		More than EAR	11	65				
Calcium		Less than EAR	54	7	0.095	0.247	(-0.016-0.510)	72.9
		More than EAR	16	8				
Magnesium		Less than EAR	43	6	0.003	0.255	(0.034-0.475)	65.9
		More than EAR	23	13				
Iron		Less than EAR	55	7	0.046	0.123	(-0.166-0.412)	70.6
		More than EAR	18	5				
Vitamin A		Less than EAR	27	14	0.017	-0.045	(-0.255-0.164)	47.1
		More than EAR	31	13				
Vitamin B1		Less than EAR	40	14	0.719	0.196	(-0.029-0.422)	63.5
		More than EAR	17	14				
Vitamin B2		Less than EAR	30	12	0.833	0.295	(0.092-0.498)	64.7
		More than EAR	18	25				
Vitamin B6		Less than EAR	20	15	0.324	0.128	(-0.084-0.339)	56.5
		More than EAR	22	28				
Vitamin B12		Less than EAR	1	47	<0.001	-0.116	(-0.386-0.154)	9.4
		More than EAR	30	7				
Vitamin C		Less than EAR	36	11	0.015	0.058	(-0.165-0.281)	55.3
		More than EAR	27	11				

## Discussion

This study demonstrated that a mobile application (Calomeal®) can provide nutrient intake estimates highly comparable to those obtained from traditional weighed food records among Japanese female university students. The Spearman correlation coefficients between the mobile application and the weighed food records were high for most nutrients (ρ≥0.7), indicating strong agreement, although moderate correlations were observed for certain micronutrients such as magnesium, iron, and vitamin B12 at specific meals.

Several previous studies have evaluated the validity and feasibility of mobile dietary assessment tools. Shinozaki and Murakami found that five major Japanese diet-tracking apps, including Calomeal, showed moderate to high correlations (ρ=0.60-0.85) with traditional weighed food records for energy and nutrient intakes at the population level [8]. Internationally, Sharp and Allman-Farinelli [5], Ambrosini et al. [7], and Zhang et al. [10] reported similar findings, with mobile applications providing acceptable estimates of dietary intake compared to 24-hour recalls or traditional weighed food records, although absolute values for some nutrients (notably fat and micronutrients) were sometimes underestimated. Our analysis also showed that the prevalence of EAR inadequacy, as assessed by the mobile application, closely mirrored that determined by the weighed food records for most nutrients. The agreement rates (kappa coefficients) for classifying participants as above or below the EAR were moderate to high for several nutrients, particularly calcium, vitamin B2, and vitamin B12, but were lower for iron and some other micronutrients. These results suggest that mobile applications can be reliable tools for population-level dietary assessment, especially in estimating the prevalence of nutrient inadequacy.

In contrast, the agreement between the mobile application and FFQ was substantially lower, both in terms of absolute nutrient intake and in classifying EAR inadequacy. The correlation coefficients between the weighed food records and FFQ were low for most nutrients, and kappa statistics indicated poor agreement for nutrient adequacy classification. This is in line with earlier reports indicating that FFQs, while useful for ranking individuals by intake, are less accurate for estimating absolute intake or prevalence of inadequacy, particularly for micronutrients [17]. Similar trends have been observed in both Japanese and international cohorts, where FFQs tend to underestimate or overestimate certain nutrients compared to more detailed records or recalls [10,18]. FFQs are widely accepted as alternative indicators of nutrient intake in conventional nutritional epidemiology studies; however, they are not suitable for use in dietary surveys that aim to accurately measure nutrient intake, such as the National Health and Nutrition Examination Survey [19].

The strengths of mobile applications include reduced participant burden, real-time data entry, and automated nutrient analysis, which collectively enhance feasibility for large-scale or long-term studies. Therefore, it may also be possible to use it to study habitual eating habits in individuals [20]. In contrast, traditional weighed food records, while highly accurate, are labor-intensive and may not be practical for large cohorts or repeated measurements [21,22]. Our study supports the use of mobile applications for population-based assessments, particularly when estimating the proportion of individuals below the EAR, a key metric in Japanese nutritional epidemiology.

However, several limitations should be noted. First, while the mobile application performed well for most nutrients, agreement was lower for certain micronutrients, such as iron, likely due to limitations in food composition databases or challenges in estimating portion sizes for mixed dishes. Second, the study population consisted of young, female, nutrition-trained participants, which may limit generalizability. Further research is needed to validate these findings in other demographic groups, including older adults, children, and individuals with chronic health conditions. Additionally, the study did not directly assess user burden or acceptability of the mobile application compared to traditional weighed food records or FFQ, which could influence compliance and data quality in real-world settings.

## Conclusions

This study demonstrates that mobile applications, such as Calomeal, are a valid and practical alternative to traditional weighed food records for dietary assessment in Japan. The app showed strong correlations with weighed food records for most nutrients, indicating high accuracy, while also offering significant advantages in terms of convenience and reduced participant burden. In contrast, the FFQ exhibited weaker correlations and lower agreement with both weighed records and app-based assessments. These findings suggest that mobile applications can enhance the feasibility of large-scale nutritional epidemiology studies by streamlining data collection and improving precision. Further research is warranted to evaluate their effectiveness across diverse populations and to explore long-term usability in various demographic groups.

## References

[1] Koshida E, Okada C, Okada E, Matsumoto M, Murai U, Takimoto H (2019). Comparison of national nutrition surveys between Japan and other countries [Article in Japanese]. Eiyo Gakuzashi.

[2] Gibson RS, Charrondiere UR, Bell W (2017). Measurement errors in dietary assessment using self-reported 24-hour recalls in low-income countries and strategies for their prevention. Adv Nutr.

[3] Ikeda N, Takimoto H, Imai S, Miyachi M, Nishi N (2015). Data resource profile: the Japan National Health and Nutrition Survey (NHNS). Int J Epidemiol.

[4] Shim JS, Oh K, Kim HC (2014). Dietary assessment methods in epidemiologic studies. Epidemiol Health.

[5] Sharp DB, Allman-Farinelli M (2014). Feasibility and validity of mobile phones to assess dietary intake. Nutrition.

[6] Hutchesson MJ, Rollo ME, Callister R, Collins CE (2015). Self-monitoring of dietary intake by young women: online food records completed on computer or smartphone are as accurate as paper-based food records but more acceptable. J Acad Nutr Diet.

[7] Ambrosini GL, Hurworth M, Giglia R, Trapp G, Strauss P (2018). Feasibility of a commercial smartphone application for dietary assessment in epidemiological research and comparison with 24-h dietary recalls. Nutr J.

[8] Shinozaki N, Murakami K (2020). Evaluation of the ability of diet-tracking mobile applications to estimate energy and nutrient intake in Japan. Nutrients.

[9] Shinozaki N, Murakami K, Masayasu S, Sasaki S (2023). Usual nutrient intake distribution and prevalence of nutrient intake inadequacy among japanese children and adults: a nationwide study based on 8-day dietary records. Nutrients.

[10] Zhang L, Misir A, Boshuizen H, Ocké M (2021). A systematic review and meta-analysis of validation studies performed on dietary record apps. Adv Nutr.

[11] Kusano Y FK, Yamaguchi M, Sugawara M, Tamano M (2022). Dietary counseling based on artificial intelligence for patients with nonalcoholic fatty liver disease. Artificial intelligence in gastroenterology.

[12] Tsunemi A, Sato J, Sugimoto S (2021). A pilot study of intervention with a mobile application visualizing the macronutrient content for type 2 diabetes at a Japanese center. J Clin Med Res.

[13] (2025). Ministry of Education, Culture, Sports, Science and Technology, Japan: Japanese Food Standard Ingredients Table (eighth revision) supplemented 2023 [Webpage in Japanese]. https://www.mext.go.jp/a_menu/syokuhinseibun/mext_00001.html.

[14] Yokoyama Y, Takachi R, Ishihara J (2016). Validity of short and long self-administered food frequency questionnaires in ranking dietary intake in middle-aged and elderly Japanese in the Japan Public Health Center-based prospective study for the next generation (JPHC-NEXT) protocol area. J Epidemiol.

[15] (2019). Dietary Reference Intakes for Japanese (2020 Edition): Report of the Committee to Establish "Dietary Reference Intakes for Japanese" [Book in Japanese].

[16] Murphy SP, Guenther PM, Kretsch MJ (2006). Using the dietary reference intakes to assess intakes of groups: pitfalls to avoid. J Am Diet Assoc.

[17] Iizuka K, Ishihara T, Watanabe M (2022). Nutritional assessment of hospital meals by food-recording applications. Nutrients.

[18] Bingham S, Luben R, Welch A, Low YL, Khaw KT, Wareham N, Day N (2008). Associations between dietary methods and biomarkers, and between fruits and vegetables and risk of ischaemic heart disease, in the EPIC Norfolk cohort study. Int J Epidemiol.

[19] (2025). Health Japan 21 Analysis and Assessment Project: National Health and Nutrition Survey. https://www.nibn.go.jp/eiken/kenkounippon21/en/eiyouchousa/.

[20] Lieffers JR, Hanning RM (2012). Dietary assessment and self-monitoring with nutrition applications for mobile devices. Can J Diet Pract Res.

[21] Kwan ML, Kushi LH, Song J (2010). A practical method for collecting food record data in a prospective cohort study of breast cancer survivors. Am J Epidemiol.

[22] Ortega RM, Pérez-Rodrigo C, López-Sobaler AM (2015). Dietary assessment methods: dietary records. Nutr Hosp.

